# Hyperspectral imaging and K-means clustering for material structure classification and detection of unmanned aerial vehicles

**DOI:** 10.1038/s41598-025-16205-z

**Published:** 2025-08-24

**Authors:** Amr Saber, Alaaeldin Mahmoud, Yasser H. El-Sharkawy

**Affiliations:** Optoelectronics and Automatic Control Systems Department, Military Technical College, Kobry El-Kobba, Cairo, Egypt

**Keywords:** Detection of UAVs, Carbon fiber composite, Glass fiber composite, Spectral signatures, Hyperspectral imaging, K-Means clustering, Materials science, Techniques and instrumentation

## Abstract

Unmanned aerial vehicles (UAVs) have become increasingly widespread in a variety of industries due to their versatility and efficiency in applications such as agriculture, surveillance, logistics, and construction. However, their rapid adoption has introduced challenges related to detection and classification, especially in the context of privacy, public safety, and national security. Conventional UAV detection methods, such as radar, thermal imaging, and acoustic systems, face limitations in accurately distinguishing between UAVs and other airborne objects. Additionally, these systems often fail to differentiate between UAVs constructed from different materials, such as carbon fiber-reinforced polymers (CFRP) and glass fiber-reinforced polymers (GFRP), which significantly affect the UAV’s radar and thermal profiles. This paper presents a promising approach for UAV detection based on the material composition of their structures using hyperspectral imaging (HSI) and K-Means (K-M) clustering. Using the proposed approach, we found that CFRP can be detected at 700 nm. While GFRP can be detected at 530 nm. By applying the K-M clustering algorithm to the spectral data, we successfully classify these materials without prior knowledge of object types. The proposed method shows high effectiveness in accurately distinguishing between UAVs based on their material composition, offering improvements over traditional detection methods that rely on shape, size, or heat signatures. This research contributes a new dimension to UAV detection by focusing on material-specific classification, providing significant potential for applications in security and surveillance, where understanding the structural composition of a UAV is critical for effective identification and mitigation strategies.

## Introduction

In recent years, UAVs, also known as drones, have become increasingly prevalent across a wide range of industries due to their versatility in applications such as traffic surveillance^1,2^, movie production^3^, disaster management^4,5^, search and rescue^6,7^, goods delivery^8,9^, agriculture^10^, and construction^11^. Their ability to operate autonomously and efficiently has made them indispensable tools, particularly in sectors requiring high operational efficiency, safety, and sustainability^12,13^. In agriculture, drones are employed for crop monitoring and precision farming, while in construction, they enhance project oversight and site management. As drone technology continues to evolve, their applications are expected to expand, offering innovative solutions to traditional industrial challenges. Despite their advantages, UAVs pose significant risks^14–16^, particularly regarding security and privacy^17,18^. Drones can easily breach restricted airspaces, posing threats to critical infrastructure, including airports, power plants, and military installations. Additionally, drones can be exploited for malicious purposes, such as surveillance or delivering harmful payloads^19^. A fundamental requirement for effective direct defense against attacking UAVs is their detection^20–23^. However, several key challenges must be considered in UAV detection:

- UAVs are often difficult to distinguish from other small flying objects, such as birds, necessitating new techniques to reduce false alarm rates and improve detection accuracy^24–26^.
- Their small size and agility, combined with rapid maneuverability, make them hard to detect with conventional methods, resulting in weak reflected signals that can easily be lost in environmental noise^27^.
- Additionally, the lack of prior information about non-cooperative UAVs—such as model, size, and other specifications—further complicates accurate detection^28^.

Given these challenges, the growing ubiquity of UAVs has made their detection and classification a critical area of research, particularly for applications related to national security, privacy protection, and public safety. Effective UAV detection is an essential foundation for implementing countermeasures against unauthorized or malicious drone activity^29–31^. Traditional detection methods face significant challenges in identifying UAVs effectively, particularly due to the complex environmental and operational conditions drones encounter^32–34^. These limitations highlight the need for advanced detection techniques tailored to the unique characteristics and behaviors of UAVs. A critical aspect of UAV detection is understanding the material composition of UAVs, which directly influences their flight performance, stealth characteristics, and overall detectability^35–40^. Most modern UAVs are constructed from composite materials, such as CFRP and GFRP, which are used in various components such as fuselages, wings, and landing gear^41–46^. The ability to classify these materials accurately is vital, as they impact the UAV’s radar cross-section, thermal profile, and overall detectability by sensors. Several methods have been developed for UAV detection, including radar-based, radio frequency (RF)-based, video-based detection, and thermal imaging systems. While radar systems excel in long-range detection and speed measurement, they often fail to distinguish between UAVs and birds due to similar radar cross-Sects.^47,48^. RF-based detection systems, which monitor communication signals between the UAV and its controller, are effective but limited to detecting drones actively transmitting signals and not suitable for autonomous drone detection^49–51^. Video-based detection, which relies on analyzing surveillance images captured by cameras, offers advantages such as medium detection range, accurate localization, low cost, and straightforward human interpretation. However, its effectiveness significantly decreases at night and in low-visibility conditions^52,53^, This limitation can be addressed by fusing visible spectrum and infrared thermal imaging. Nonetheless, a major challenge remains: the inability to reliably differentiate between drones and birds, leading to a high rate of detection errors^54^. Thermal imaging is particularly useful in detecting heat signatures but is less effective in identifying UAVs constructed from low-heat-emitting materials, such as electric drones with plastic bodies^55,56^. Given these limitations in existing detection methods, the motivation behind this research is to address these shortcomings by introducing a promising approach based on hyperspectral imagery^57–59^ to analyze and classify the material composition of UAVs. By focusing on the spectral characteristics of materials, this study seeks to improve the accuracy of UAV detection, especially in challenging environments with varying illumination and noise conditions. However, applying this technique for UAV material classification is still in its early stages of development^60,61^, and there is a significant gap in leveraging image clustering techniques, such as K-M clustering^62,63^, to classify UAV materials in complex environments. This paper presents a cutting-edge approach for UAV detection and classification, focusing on the material composition of UAV structures using hyperspectral imagery (400–1000 nm). By capturing the spectral signatures of commonly used UAV materials, such as CFRP and GFRP composites, we apply K-M clustering to group UAVs based on their material composition. This allows for more accurate UAV detection, especially in scenarios where other detection methods fall short due to environmental noise or the inability to distinguish UAVs from other small flying objects. The contributions of this work are as follows:

- A pilot study of a hyperspectral imagery-based method for discriminating between UAVs made from CFRP and GFRP composites.
- Application of K-M clustering to classify UAV materials based on spectral signatures, improving detection accuracy and reducing false positives.

This method also demonstrates the ability to distinguish UAV structural materials from other airborne objects. Each material exhibits a distinct hyperspectral reflectance signature, determined by its unique chemical composition and microstructural properties. These spectral characteristics differ fundamentally from those of organic or metallic targets. In this study, the effectiveness of the approach was validated through a real-world test involving a retrieved UAV fragment that was visually indistinguishable from other debris. By applying the same HSI and K-M clustering workflow, the unknown sample was consistently grouped with the reference cluster of GFRP, without the need for any destructive testing. Subsequent laboratory analysis confirmed the fragment’s fiberglass composition, validating the reliability of this non-contact, spectral-based method in identifying UAV materials. This research was intentionally designed as a proof-of-concept to demonstrate that hyperspectral signatures, when combined with unsupervised K-M clustering, can effectively differentiate between CFRP and GFRP in a controlled environment. A uniform black background was used to isolate the intrinsic reflectance properties of the materials, allowing for clear spectral clustering. However, it is recognized that real-world UAV detection must address more complex scenarios involving diverse backgrounds such as vegetation, urban structures, airborne dust, and variable lighting. To overcome these challenges, future work will focus on extending the validation process to include textured backgrounds, natural sunlight, and dynamic flight conditions, thereby enhancing the system’s applicability in operational environments. The proposed HSI technique provides a powerful tool for discriminating between different materials based on their spectral signatures. This makes HSI an attractive solution for UAV detection and classification, particularly when enhanced by advanced image clustering algorithms like K-M clustering. This approach is particularly relevant for applications involving security, surveillance, and critical infrastructure protection, where understanding the material structure of a UAV can improve detection and response strategies.

## Materials and methods

**Fig. 1:**
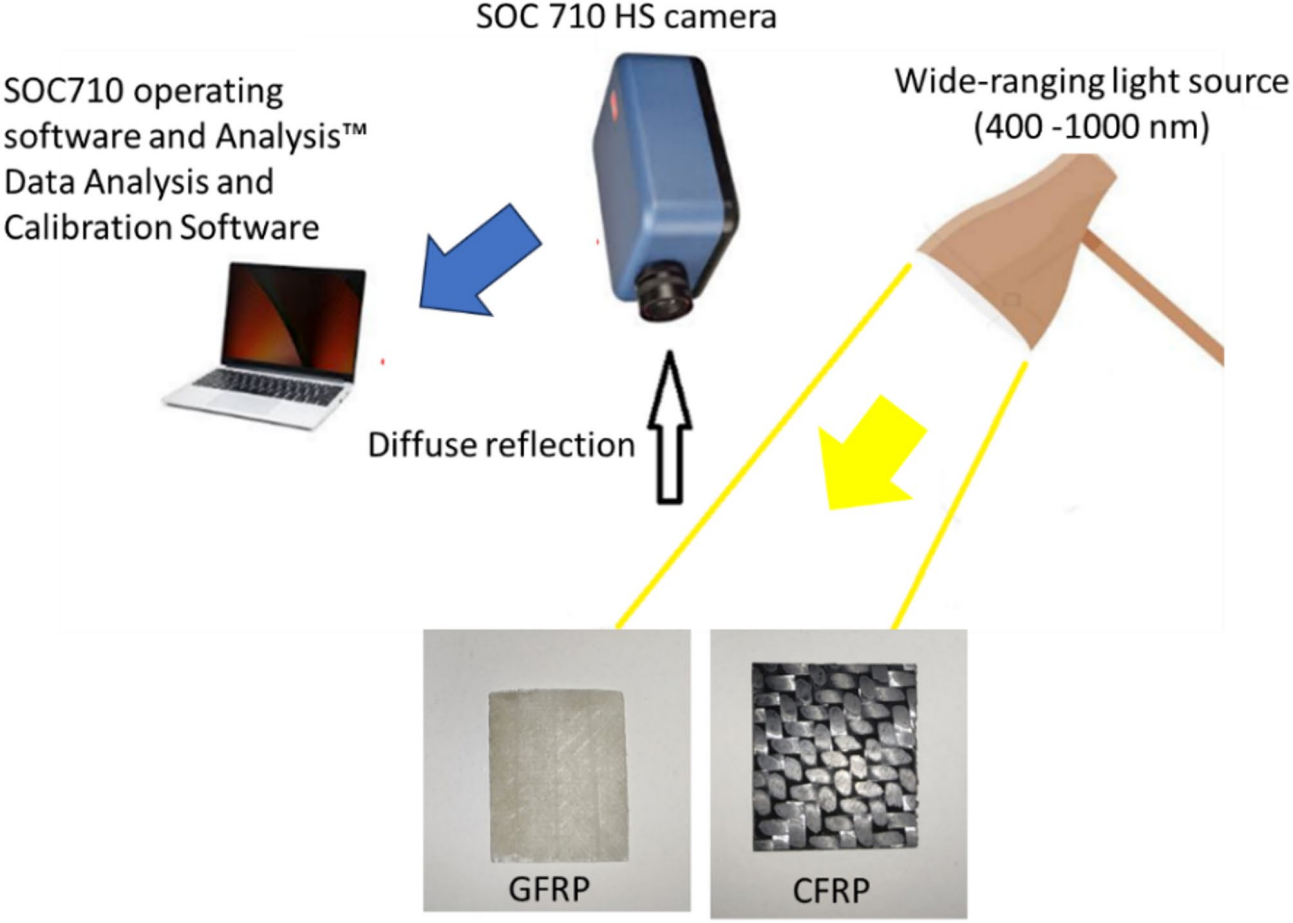
Diagram of the Lab HSI system setup.

This section outlines the materials and techniques used in this study to examine CFRP and GFRP composite materials and describes how our imaging method was implemented to achieve the intended results. The experimental setup for optical scanning is illustrated in Fig. 1. A broad-spectrum light source (lamp with a wavelength range of 400–1000 nm, 20 watts) was used to illuminate the samples, ensuring that the selected wavelength range aligns with the imaging capabilities of our HSI camera and enhances the applicability of our findings. When light is directed onto the sample materials (carbon fiber and fiberglass composites), part of it interacts with the surface and undergoes absorption or scattering within the CFRP and GFRP samples. This process is known as diffuse reflectance, where the incident light is reflected in multiple directions^64–66^. The reflected light is then captured by the proposed HSI camera (SOC710, Surface Optics Corporation), which records spectral signatures unique to each material. The captured data is subsequently transmitted to a computer equipped with analysis software, including the SOC710 operating software and Analysis™ (HS-Analysis 2XL, https://www.surfaceoptics.com), for further processing and evaluation.

### Sample preparation

**Fig. 2:**
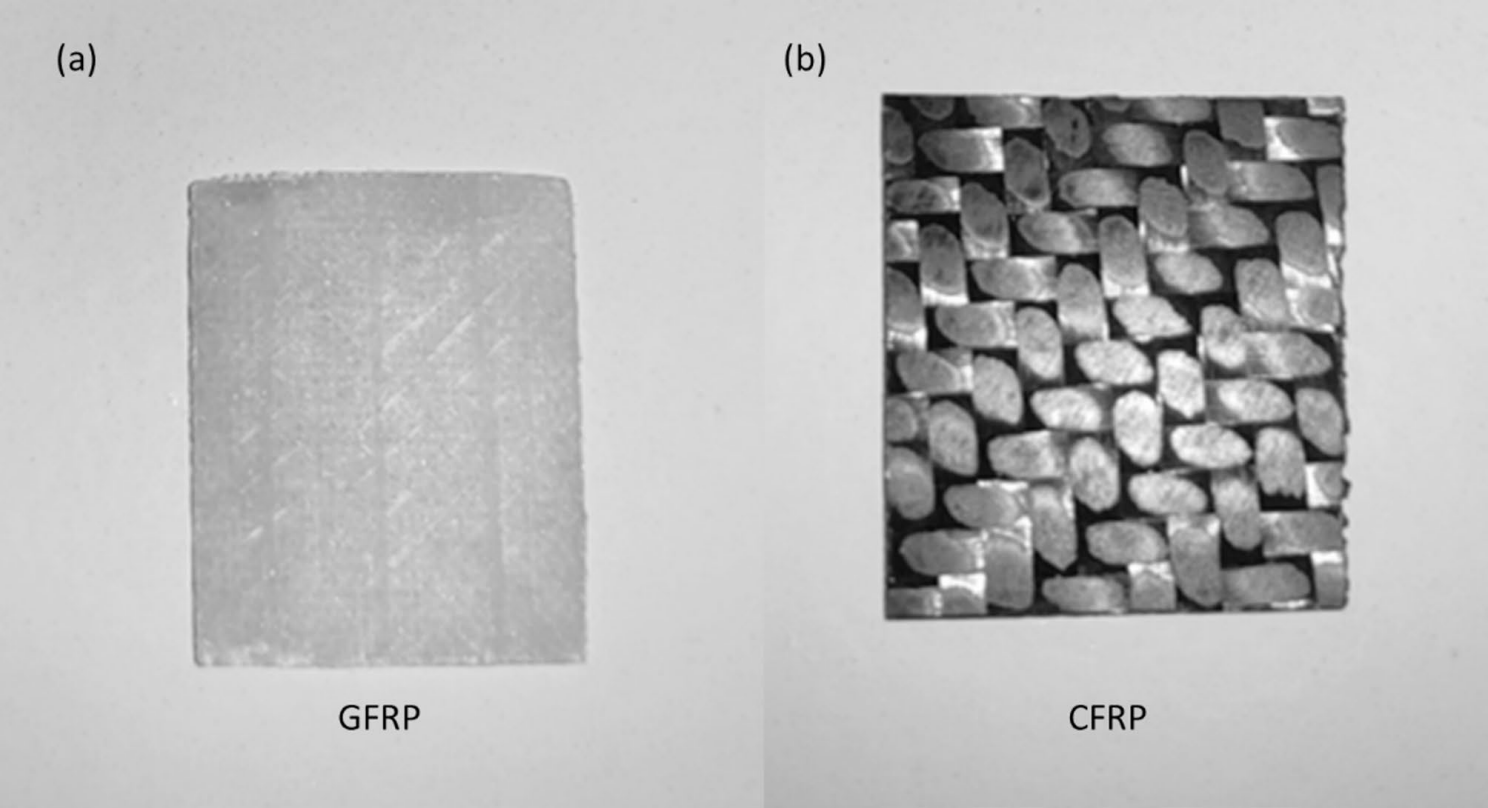
(**a**) Fiberglass-reinforced polymer (GFRP) sample #1; (**b**) Carbon fiber-reinforced polymer (CFRP) sample #1.

In this investigation, a total of twenty composite material samples were prepared and analyzed: ten GFRP samples and ten carbon fiber-reinforced polymer CFRP samples. Each sample was prepared with dimensions of 20 mm × 15 mm to ensure uniformity in imaging and analysis. Each GFRP sample consisted of epoxy resin reinforced with E-glass fiber, fabricated as four stacked layers: layer 1 (400 g/m², + 45°/-45°), layer 2 (400 g/m², 0°/90°), layer 3 (100 g/m², 0°/90°), and layer 4 (25 g/m², 0°/90°), with a total thickness of approximately 0.5 mm, as shown in Fig. 2 (a). Each CFRP sample was fabricated using 2 × 2 twill weave, 3k black carbon fiber cloth (200 g/m²) with epoxy infusion and a 0°/90° fiber orientation, yielding a total thickness of 0.6 mm, as shown in Fig. 2 (b). To ensure statistical reliability and to evaluate the sensitivity of the clustering-based classification method, each of the twenty samples was imaged ten times under identical lighting and imaging conditions using the HSI setup described in Sect. 2.2. This resulted in a dataset of 200 HS images (100 for GFRP and 100 for CFRP), which enabled the computation of detection sensitivity, and other performance metrics. Repeating measurements across multiple trials also accounted for minor variations in positioning, and surface texture, thereby ensuring robust validation of the proposed segmentation and material classification methodology.

### HS imager arrangement

**Fig. 3:**
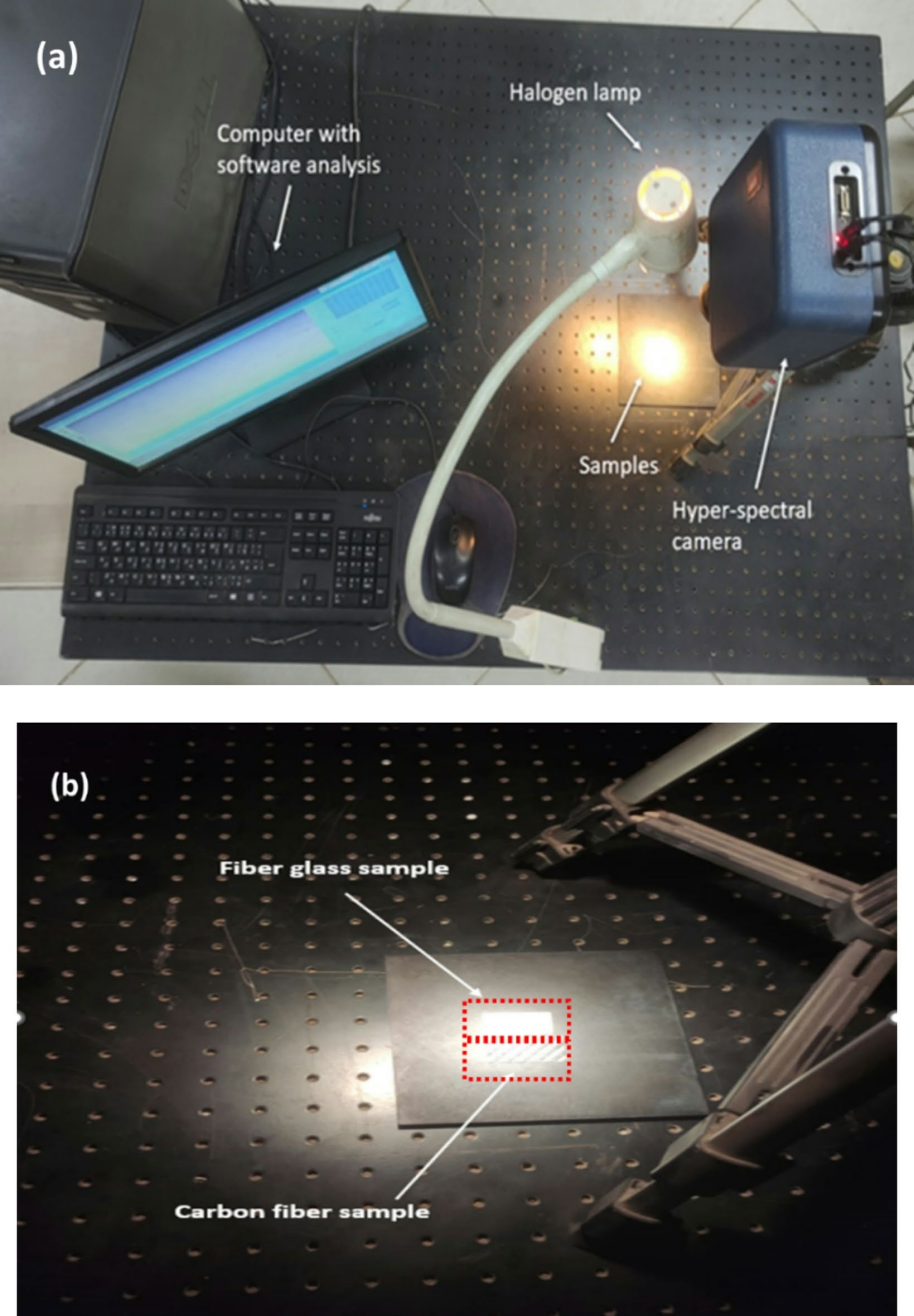
(**a**) Benchtop HS imagery system setup for examining composite samples (CFRP and GFRP); (**b**) Close-up view of samples undergoing analysis.

For this study, we utilized the SOC710 HSI camera which provides a spectral resolution of approximately 5 nm. Each of the 128 frames in the captured spectral cube image represents a single wavelength. A line-scanning HSI camera with a resolution of 520 pixels per line and approximately 696 lines per cube was used to capture HS images of both the examined fiberglass and carbon fiber samples (GFRP and CFRP). Operating in standard lighting conditions, the camera captures images across the spectral range of 0.35 to 1.0 microns. The imaging setup included a Schneider Xenoplan optical lens with a 35 mm focal length. To ensure uniform illumination, the camera’s focal point was carefully aligned with the light source, and both the utilized camera and composite samples were positioned to maintain consistent optical paths and even light distribution throughout the experiment. The detector integration time and light source intensity were adjusted to prevent saturation. Image acquisition was conducted with the camera positioned perpendicular to the optical bench at a 0° nadir angle. The SOC710 operating software, along with HSAnalysis™ Data Analysis and Calibration Software (HS-Analysis 2XL, https://www.surfaceoptics.com), was used for data acquisition, exposure control, and managing the motorized linear scanner. With a 10° field of view, the system ensured precise alignment between the composite samples and the HS imager. The light source was positioned approximately 55 cm from the tested samples, and the SOC710 HSI camera was placed around 50 cm above the optical bench, as shown in Fig. 3(a), and (b).

As depicted in Fig. 3, The proposed setup employs a benchtop halogen light source and a neutral black background to establish a clear proof-of-concept for HSI, material-based UAV classification. The static, repeatable experiment allows us to isolate and characterize the intrinsic spectral signatures of CFRP and GFRP without confounding environmental variables. By demonstrating that these materials form well-separated clusters under idealized conditions, we establish the foundational feasibility of the presented approach. Importantly, the use of a broadband halogen lamp provides a stable, uniform illumination across the 400–1000 nm range, closely approximating the spectral content of sunlight while maintaining controlled intensity for quantitative analysis. The black background minimizes unwanted reflectance, ensuring that clustering thresholds derive solely from sample properties rather than scene clutter.

### Image processing and analysis

**Fig. 4:**
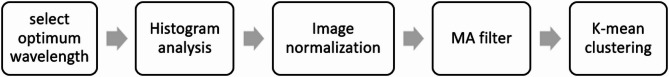
Image analysis and processing steps for enhancing the detection of drone composite materials.

To begin, identifying the optimal wavelength for each sample using the output data from the SOC710 operating software was implemented. Building on this, an advanced approach that integrates the K-M clustering algorithm for image segmentation with image enhancement techniques, including normalization and moving average (MA) filtering. This combined methodology aims to improve the analysis and classification of composite materials (CFRP and GFRP) based on their spectral properties after capturing and calibrating HS images, as illustrated in Fig. 4.

As shown in Fig. 4, once the optimal wavelength is selected for each sample, HS images of the composite samples, illuminated by a halogen lamp, undergo histogram analysis. This analysis helps assess the dispersion of pixel brightness across various spectral wavelengths. The results of this histogram analysis are critical for determining the optimal number of clusters to be used in the subsequent proposed K-M clustering process. Following this, the intensity values of all pixels are normalized to a standard scale through image normalization, ensuring uniformity across the dataset. Image normalization standardizes pixel intensities, commonly through min–max normalization, which rescales values *x* from the range $\:\left[{x}_{min},{x}_{max}\right]$ to a new range [a, b] using the formula^67^:

1 $$\:{x}^{{\prime\:}}=a+\:\frac{\left(x-{x}_{min}\right)(b-a)}{{x}_{max}-{x}_{min}}$$

Where *x* is the original pixel or the reflectance value, *x*_*min*_, *x*_*max*_ are min and max values in the dataset or spectral band, [a, b] is the new target range, and *x*′ is the normalized value. After normalization, the data is further processed using MA filter. This filter smooths the intensity values, reducing noise and random fluctuations in pixel data, while preserving the essential spectral features and patterns. This step enhances spectral consistency and preserves material-specific trends. The MA filter operates on the normalized spectral data *x*′ across the wavelength dimension. For a given spectral vector *x*′(λ), the smoothed value at wavelength index *i* is computed using^67,68^:

2 $$\:{x}_{smoothed\left({\lambda\:}_{i}\right)}^{{\prime\:}}=\frac{1}{n}\sum\:_{j=i-\frac{n-1}{2}}^{i+\frac{n-1}{2}}{x}^{{\prime\:}}\left({\lambda\:}_{j}\right)$$

Where *x*′(*λ*_*j*_) is the normalized reflectance at wavelength *λ*_*j*_, n is the window size, *λ*_*i*_ is the central wavelength for which the smoothing is being computed, and the summation includes the n wavelengths centered around *λ*_*i*_. In this study, we used *n* = 5, which averages each reflectance value with its two preceding and two succeeding neighboring wavelengths. This suppresses spectral noise while maintaining the overall shape and discriminative features of the reflectance curves necessary for accurate material classification. Furthermore, this preprocessing step is crucial for enhancing the accuracy of the implemented K-M clustering algorithm, enabling clearer differentiation between material types based on their unique spectral signatures. The K-M clustering algorithm, which forms the core of the final phase in our enhanced detection methodology, is employed for classifying the data. The objective of K-M is to partition the dataset into K clusters such that the within-cluster variance is minimized. This is expressed by the following cost function^69^:

3 $$\:J = \sum\limits_{{i = 1}}^{K} {\sum\limits_{{x^{\prime} \in C_{i} }} {\left\| {x^{\prime} - \mu _{i} } \right\|^{2} } }$$

Where *J* is the total within-cluster sum of squares to be minimized, K is the number of clusters, *x*′ is the normalized and smoothed spectral vector of a pixel, *C*_*i*_ is the set of pixels assigned to cluster *i*, *µ*_*i*_ ​ is the centroid (mean spectral vector) of cluster *i*, and $\:\left\| {x^{\prime} - \mu _{i} } \right\|$ denotes the Euclidean norm, representing the distance between a pixel and its cluster centroid. The clustering strategy assigns each pixel to the cluster with the nearest centroid in spectral space, effectively grouping pixels with similar spectral signatures corresponding to different material types. This method is widely recognized for its ability to handle complex patterns and is extensively used across various disciplines for efficient and accurate data classification, particularly when dealing with intricate datasets like HS images. In this study, data preprocessing and visualization steps algorithm were performed using DADiSP 6.5 (DSP Development Corporation, USA; https://www.dadisp.com), a tool well-suited for advanced signal processing applications. By combining these techniques, the presented approach facilitates more precise material identification and classification, which is crucial for applications such as the discrimination of composite materials used in UAVs using HS imaging.

## Results and analysis

**Fig. 5:**
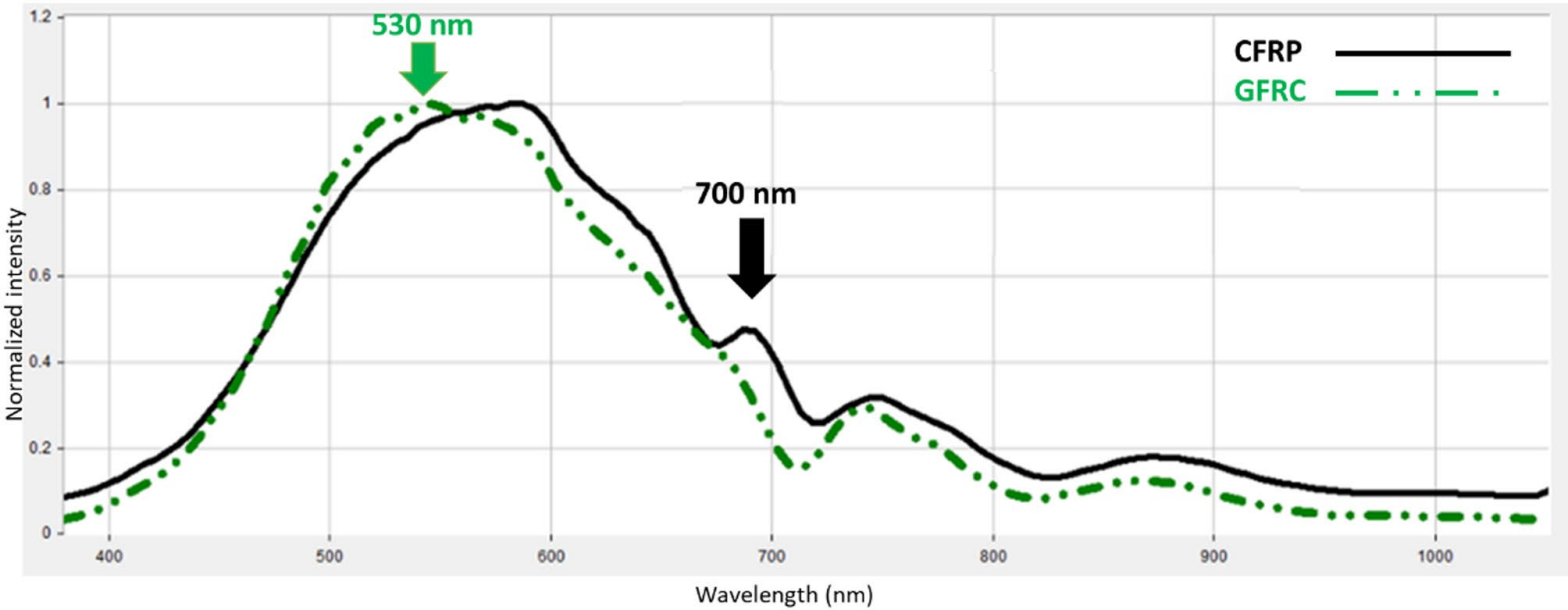
The captured normalized diffuse spectral response of the tested composite materials.

In this section, we highlight key achievements of the proposed pilot study. Through the analysis of the reflectance spectra of the studied UAVs composite materials, the experiments aim to showcase the effectiveness of the HSI technique in material identification and classification. The samples were illuminated with white polychromatic light in the wavelength range of 400 to 1000 nm. Figure 5 presents the diffuse reflectance spectra signals for the materials tested, specifically carbon fiber and fiberglass (CFRP and GFRP).

**Fig. 6:**
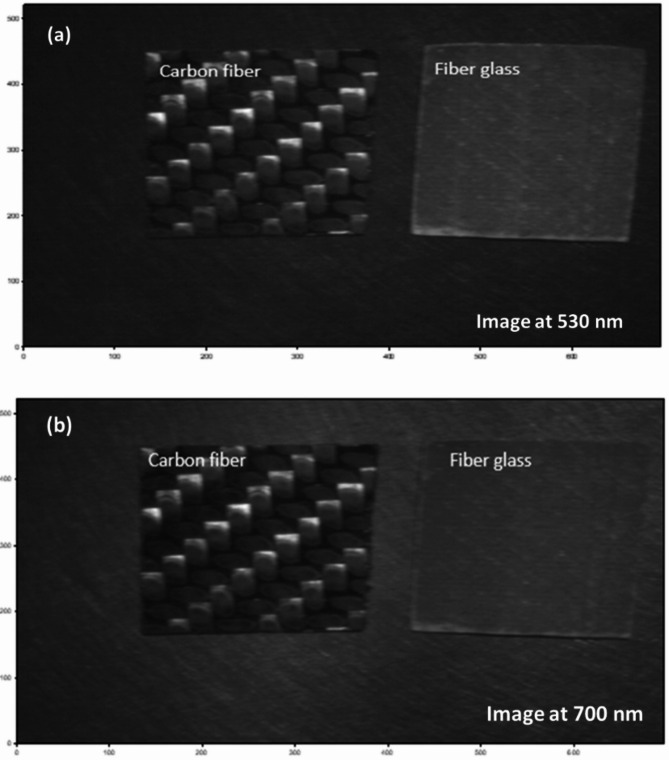
HS images captured at the two optimal wavelengths for detecting both carbon fiber and fiberglass composite materials: (**a**) at 530 nm and (**b**) at 700 nm.

As shown in Fig. 5, the normalized diffuse reflectance spectrum clearly varies depending on the material being analyzed. Additionally, specific wavelengths of the diffused reflected light are particularly effective for identifying and classifying each material. For CFRP, the most notable variation in the spectrum occurs around 700 nm, while for GFRP, the significant variation is observed near 530 nm. It is important to note that Fig. 5 displays normalized reflectance spectra, meaning that all intensity values have been scaled to a common range to remove absolute brightness effects and highlight each material’s true spectral signature. Under this normalization, although CFRP and GFRP curves may appear to be close at wavelengths like 560 nm, their spectral shapes diverge most clearly at 700 nm. At 700 nm, the normalized reflectance of CFRP exhibits a pronounced peak while GFRP remains near baseline, producing a contrast ratio of approximately 2.5:1. In contrast, the difference at 560 nm is more less, which could be obscured by sensor noise or slight illumination variances. By using normalized data, we ensure that our wavelength selection reflects intrinsic material properties rather than raw intensity artifacts, and 700 nm therefore emerges as the optimal choice for reliable CFRP discrimination. Figure 6 presents HS images captured at these optimal wavelengths, highlighting the distinct detection of both carbon fiber and fiberglass composite materials.

**Fig. 7:**
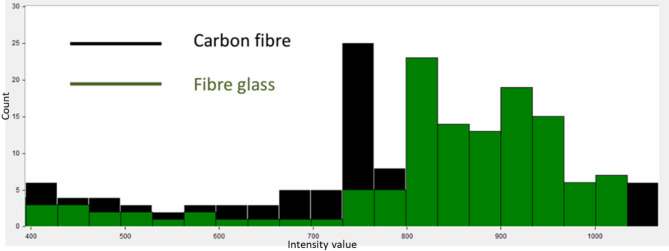
Histogram analysis for both CFRP and GFRP composite materials.

As shown in Fig. 6, while the spatial layout of the CFRP and GFRP samples is indeed the same, the images are single-band slices extracted from two distinct wavelengths (530 nm and 700 nm) selected based on spectral analysis (Fig. 5). These two wavelengths correspond to the peak spectral responses of GFRP and CFRP, respectively. At 530 nm (Fig. 6 (a)), the GFRP sample appears brighter due to higher reflectance, while the CFRP sample appears darker. Conversely, at 700 nm (Fig. 6 (b)), the carbon fiber sample becomes significantly brighter relative to the fiberglass, whose reflectance drops. This wavelength-dependent contrast difference is critical, as it forms the basis for the K-M clustering framework, which exploits these variations to isolate each material. Following this, histogram analysis is employed to examine the dispersion of pixel brightness across the full spectral range. This analysis not only provides insights into the pixel brightness distribution but also gives a comprehensive view of the data’s overall characteristics. Such information is crucial for determining the optimal number of clusters for the subsequent K-M clustering analysis, as depicted in Fig. 7. This figure illustrates the variations in pixel brightness distribution across the 400–1000 nm wavelength range, highlighting significant differences that are essential for accurate material classification.

**Fig. 8:**
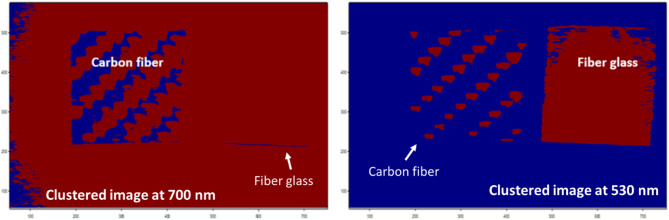
The application of the proposed K-M clustering approach using spectral images at 530 nm and 700 nm to enhance the detection of both carbon fiber (CFRP) and fiberglass (GFRP).

As shown in Fig. 7, the reflectance histograms for the two reference materials, glass fiber (GFRP, green) and carbon fiber (CFRP, black), illustrate their distinct pixel-brightness distributions across the full HS range. The vertical axis shows the number of pixels within each intensity bin. The GFRP distribution exhibits a pronounced right-skewed peak, indicating that a significant fraction of pixels reflects strongly at wavelengths where glass fiber has high reflectance (around 530 nm), whereas the CFRP histogram is narrower and left-shifted, reflecting the lower overall reflectance in accordance with its optimal response at longer wavelengths around 700 nm. Because these two histograms occupy largely distinct intensity regions with minimal overlap (non-overlapping peaks), a simple intensity threshold in the region between the two distributions can obviously separate GFRP from CFRP and background. This natural separation validates our choice of two clusters (K = 2). We conducted a sensitivity analysis across a range of candidate values (e.g., 0.2 to 0.6 for CFRP and 1.2 to 1.6 for GFRP) and evaluated the resulting segmentation performance. For CFRP, the intensity distribution consistently began separating from background noise at approximately 0.4, while for GFRP, a distinct cluster formed at intensities above 1.4. These values were adopted as thresholds in K-M clustering. Next, we applied the proposed image segmentation technique to distinguish and classify each studied composite sample at its optimal detection wavelength, as identified in the previous spectral analysis. For this purpose, we utilized the presented K-M clustering method, setting the number of clusters (K) to 2. By using a threshold value of ≥ 0.4, the K-M clustering algorithm successfully isolated CFRP from the background. Similarly, applying the K-MC algorithm with an increased threshold value of ≥ 1.4 enabled effective separation of GFRP. These segmentation results are illustrated in Fig. 8, where each material is clearly identified at its characteristic wavelength, demonstrating the precision of the proposed method for UAVs material classification.

Figure 8 demonstrates the effectiveness of the proposed approach in achieving the primary research goal: enhanced identification of drones and classification of their body materials. Our method leverages the SOC710 HS imager and a broad-spectrum light source to detect diffuse reflectance characteristics of both carbon fiber and fiberglass samples (CFRP and GFRP). By employing an advanced clustering technique that combines the K-M clustering algorithm with MA filter, we were able to clearly distinguish and classify the two materials based on their unique spectral signatures. The technique’s ability to differentiate between these materials, as shown through clustering results at the optimal detection wavelengths (530 nm for fiberglass and 700 nm for carbon fiber) highlights its precision and reliability.

**Fig. 9:**
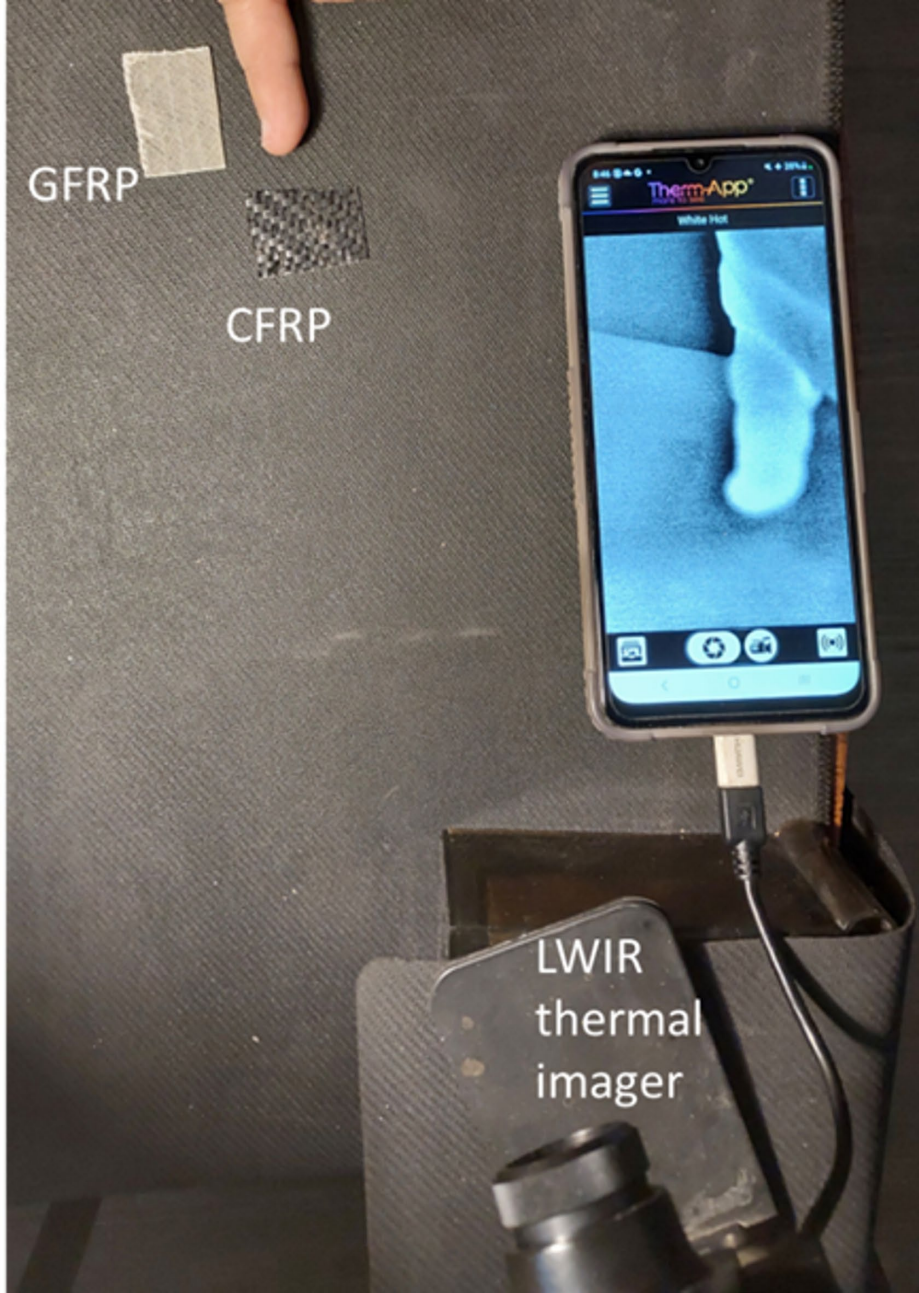
Thermal imaging with a Therm-App long-wave infrared (8–12 μm) camera failed to detect or distinguish between CFRP and GFRP samples based on their emitted heat signatures.

To quantify the effectiveness of the proposed HSI and K-M clustering approach for classifying UAV composite materials, we computed standard performance metrics based on repeated trials using labeled samples. These include True Positives (TP), False Positives (FP), False Negatives (FN), and True Negatives (TN), from which sensitivity, specificity, precision, and overall accuracy were derived. Each of the 10 CFRP and 10 GFRP samples was imaged 10 times under identical conditions, resulting in 100 classification trials for each class. The proposed method achieved a sensitivity of 95%, specificity of 93%, precision of 91%, and an overall accuracy of 94.5% in differentiating between CFRP and GFRP. Nevertheless, achieving 94.5% accuracy across 200 trials on visually similar composite materials using an unsupervised method demonstrates strong performance and validates the robustness of the proposed approach. As a benchmark, we also evaluated the detection capability of a thermal imaging method using a Therm-App long-wave infrared (LWIR) camera (8–12 μm spectral range, f = 19 mm lens), with all samples illuminated by an IR lamp. This conventional approach, which classifies UAVs based on emitted heat signatures, failed to distinguish between the CFRP and GFRP samples in all 200 trials due to the low emissivity and similar thermal behavior of the studied composite materials. These results, shown in the following figure (Fig. 9), further highlight the limitations of thermal methods and underscore the value of the discussed spectral-based technique for accurate and material-specific UAV detection. This proposed methodology shows great potential for accurate and consistent identification of drone composite materials, offering a robust solution for material classification in the field of drone and UAV technology.

## Discussion

**Fig. 10:**
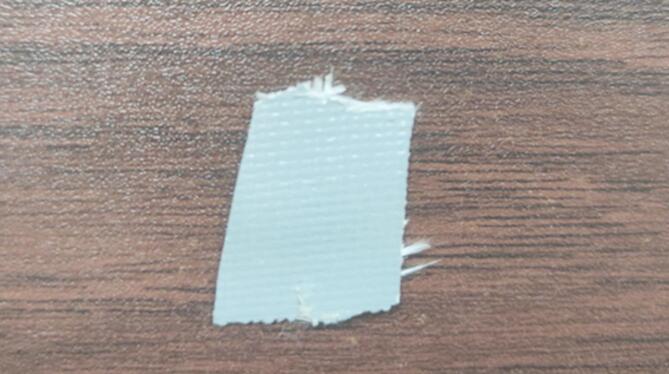
A sample retrieved from an unidentified UAV structure.

UAVs pose significant threats in security-sensitive contexts, yet they often evade conventional detection systems. Radar struggles with their small radar cross-sections and thermal imagers fail on low-emissivity composites. To address these challenges, we developed and validated an alternative non-contact, and label-free classification methodology that combines HSI with unsupervised K-M clustering. These two composites are both fiber-reinforced polymers with similar surface appearance and overlapping spectral features in certain regions. The fact that they exhibit visually indistinguishable characteristics and low thermal contrast further complicates detection using conventional systems such as RGB or thermal imagers. By capturing each material’s unique spectral fingerprint and applying data-driven clustering, this approach distinguishes between CFRP and GFRP without any prior labeling. Extensive experiments on ten samples of each material, analyzed through detailed histogram comparison and cluster validation, demonstrated robust and reproducible separation based solely on spectral signatures. This study represents a proof-of-concept framework, paves the way for material-specific UAV detection that complements existing radar and thermal methods. To validate the effectiveness of the proposed methodology for classifying unknown materials, we conducted an experiment on a real-world sample from the body of an unidentified UAV structure. As shown in Fig. 10, the sample, retrieved from the UAV, is painted, making it difficult to visually discern the underlying material. This painted surface poses challenges for traditional detection methods, which rely on external appearance or thermal profiles. Our objective was to classify this unknown sample as either fiberglass or carbon fiber using the diffuse reflection-based approach developed in this study.

**Fig. 11:**
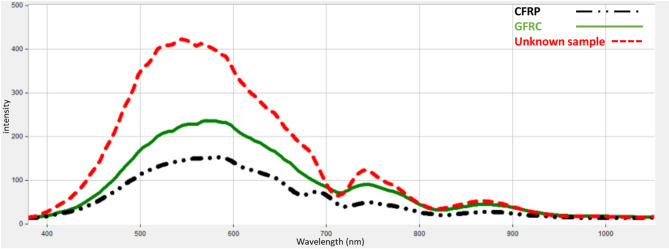
Captured diffuse spectral response of the unknown material compared to reference materials (CFRP and GFRP).

**Fig. 12:**
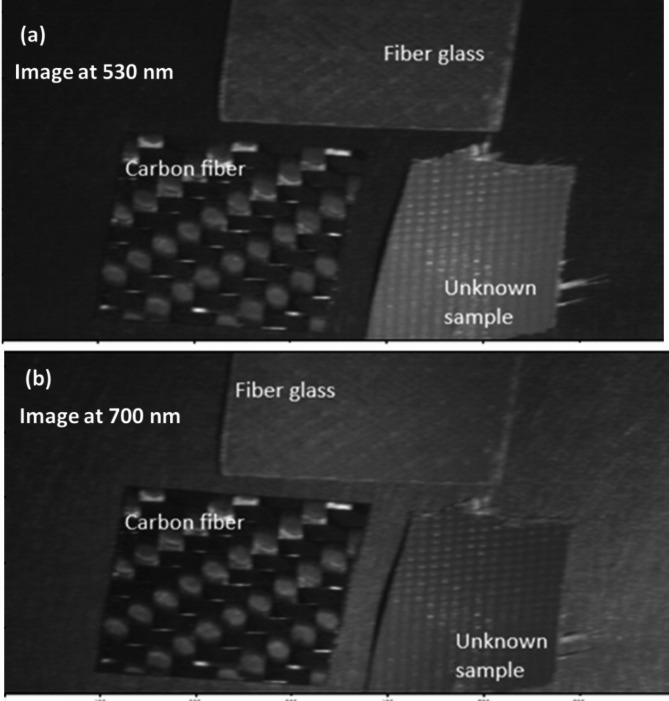
HS images of the unknown material at optimal detection wavelengths: (**a**) 530 nm and (**b**) 700 nm.

Using the SOC710 HS imager, we captured the diffuse reflectance of the unknown sample across the 400–1000 nm range. The resulting spectral data, as shown in Fig. 11, was processed to extract HS images at the optimal wavelengths identified in the proposed findings: 530 nm for fiberglass and 700 nm for carbon fiber. These wavelength-specific images are presented in Fig. 12.

**Fig. 13:**
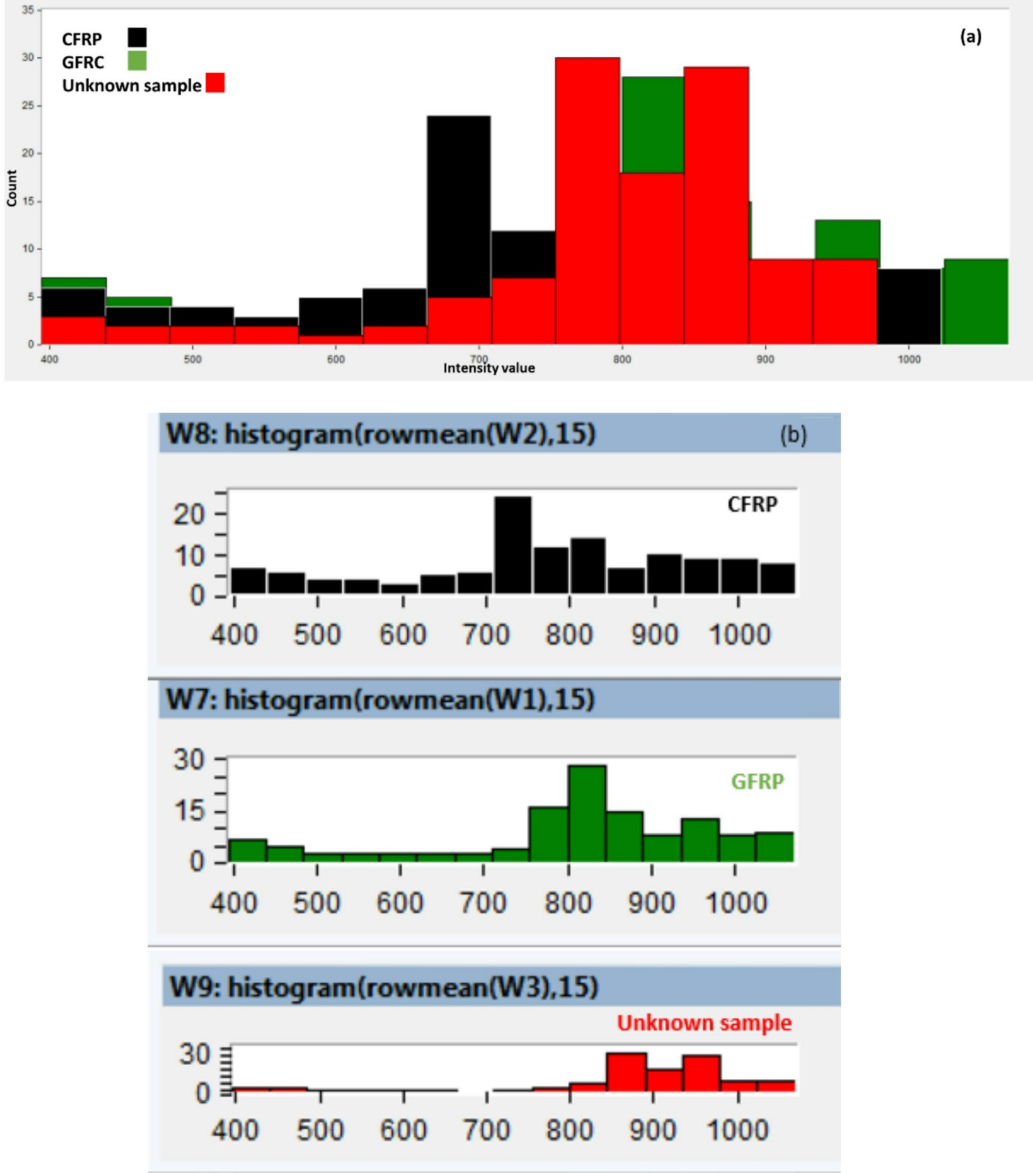
(**a**, **b**) Histogram analysis comparing the pixel brightness distribution of the unknown UAV sample with carbon fiber and fiberglass materials.

As shown in Fig. 11, The unnormalized reflectance curve of the unknown flying object sample is displayed, including the effects of surface treatment, or paint. The difference in intensity highlights the real-world challenge of spectral classification under uncontrolled conditions. Based on the framework methodology we developed, which combines wavelength selection and intensity normalization as described in Sect. 3, the unknown UAV sample could be classified. Next, histogram analysis was applied to the HS images of the unknown sample and compared its pixel brightness distribution to that of the reference materials (CFRP and GFRP). Figure 13 (a) and (b) present histogram analyses comparing the pixel intensity distributions of the unknown UAV sample with those of carbon fiber and fiberglass. The similarity in distribution patterns offers a strong preliminary indication that the unknown material closely aligns with one of the known composite classes.

**Fig. 14:**
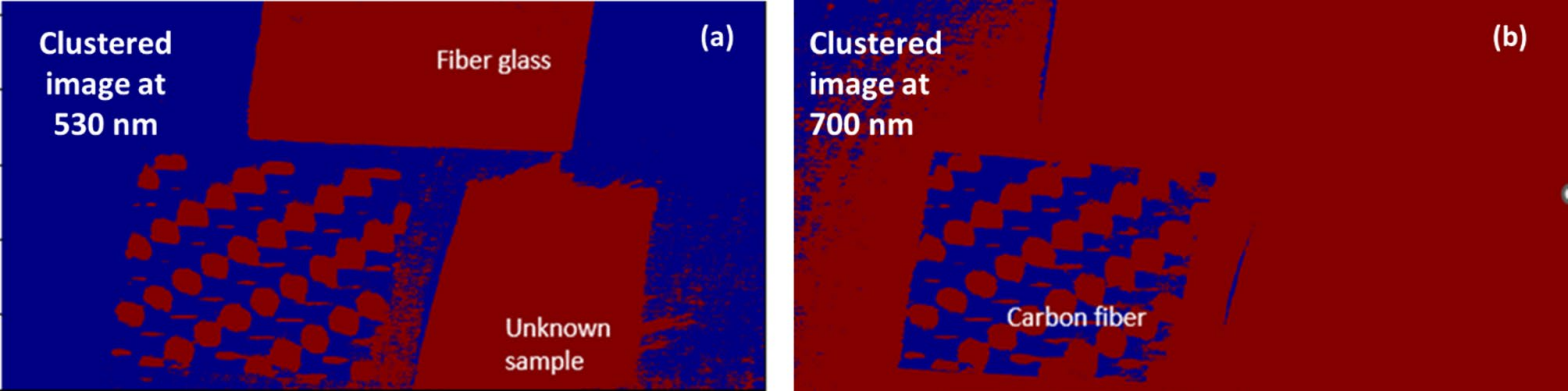
Application of the proposed K-M clustering method using HS images at (**a**) 530 nm and (**b**) 700 nm for classification of the unknown UAV material.

According to Fig. 13, The unknown sample (red) displays a peak and intensity spread that closely matches the fiberglass reference (green). This pattern suggests that the unknown material shares the optical behavior of fiberglass, rather than carbon fiber. Moreover, the close overlap in histogram bins between the unknown sample and fiberglass confirms their statistical similarity, reinforcing the material classification. To confirm the classification approach, the enhanced image processing technique using the K-M clustering method was applied, setting k = 2. By applying the threshold values of ≥ 1.4 (specific to GFRP) and ≥ 0.4 (specific to CFRP), we successfully segmented the HS images of the unknown sample. The clustering results, shown in Fig. 14, conclusively classified the unknown UAV material as fiberglass.

**Fig. 15:**
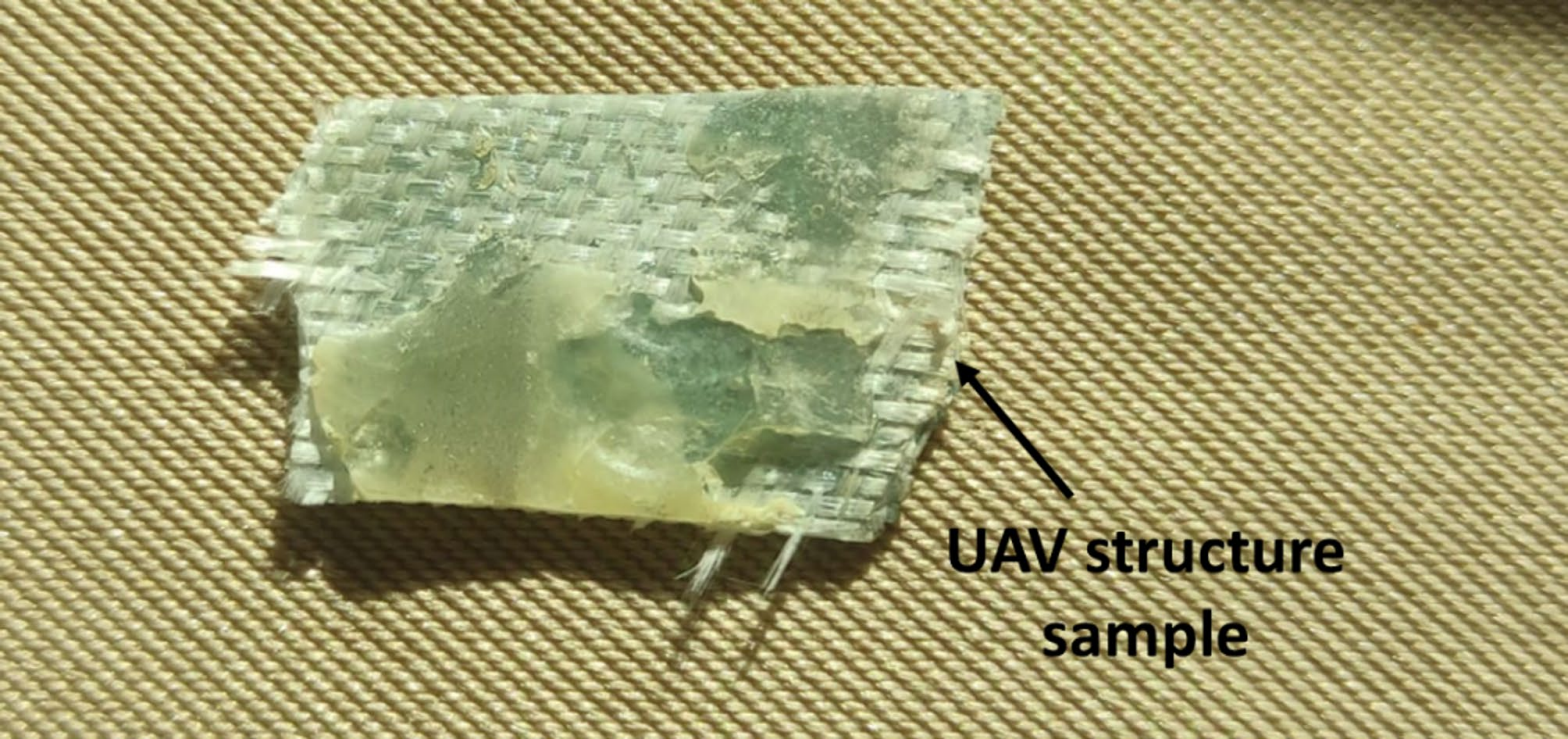
Physical analysis confirming that the UAV material is composed of GFRP.

According to Fig. 14 outcomes, the unknown sample was evaluated at both key wavelengths: 530 nm (GFRP) and 700 nm (CFRP). K-M clustering with thresholds derived from prior experiments successfully segmented the unknown sample only at 530 nm using the GFRP threshold (≥ 1.4). Clustering failed at 700 nm, even using the lower threshold for CFRP. This confirms that the material is spectrally and statistically similar to GFRP, nearly consistent with the histogram overlap. These results strongly validate the classification conclusion. The promising outcomes were further validated through a destructive examination of the UAV sample to analyze its internal structure. This physical analysis confirmed that the material is indeed composed of fiberglass, as illustrated in Fig. 15. This dual-layered verification reinforces the accuracy and reliability of our proposed methodology for UAV material classification.

**Fig. 16:**
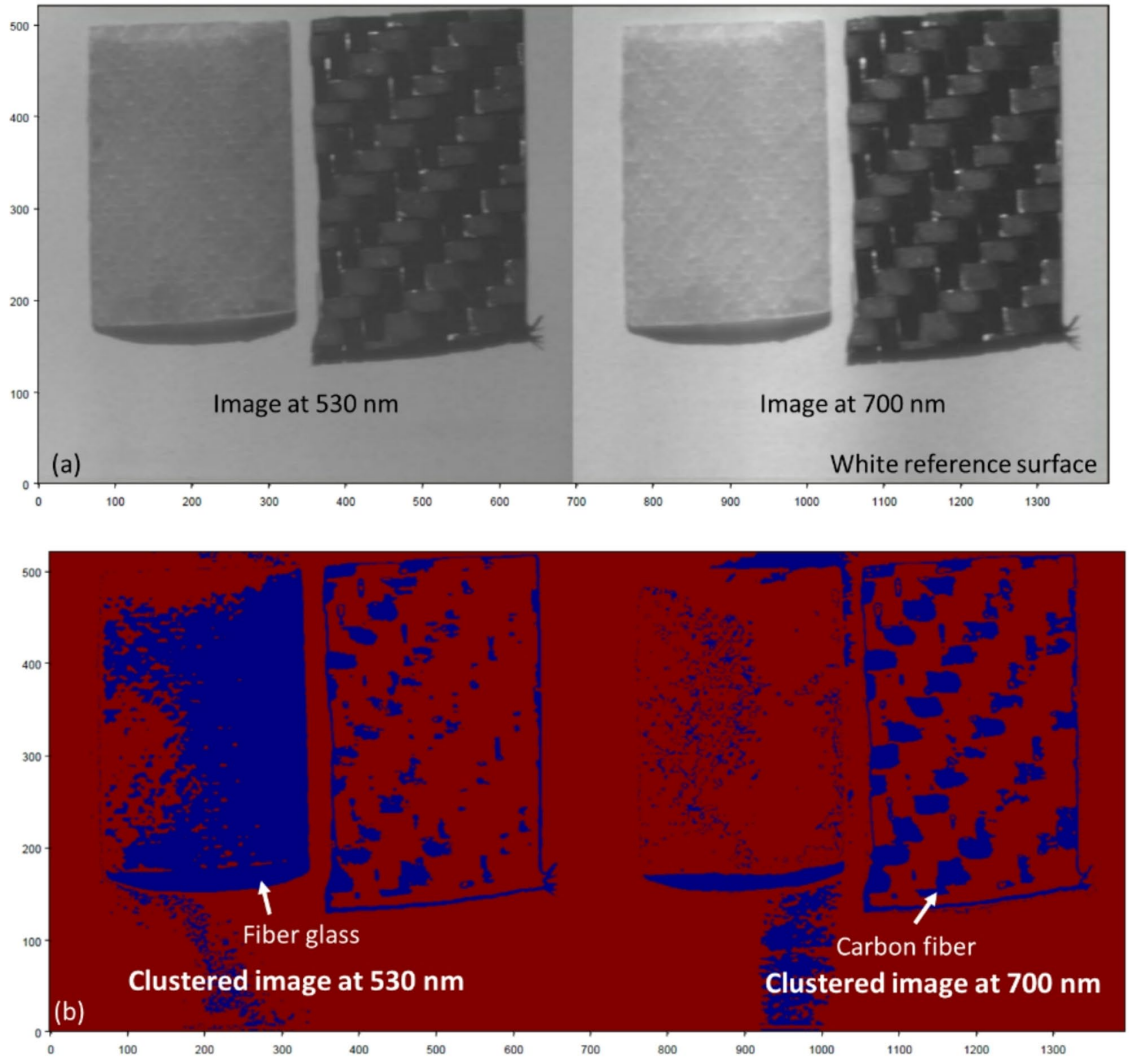
(**a**) HS images of GFRP and CFRP samples captured at 530 nm and 700 nm against a high-reflectance white reference background. (**b**) Corresponding K-M clustering segmentation results at 530 nm and 700 nm, showing clear separation of glass fiber and carbon fiber composites.

**Fig. 17:**
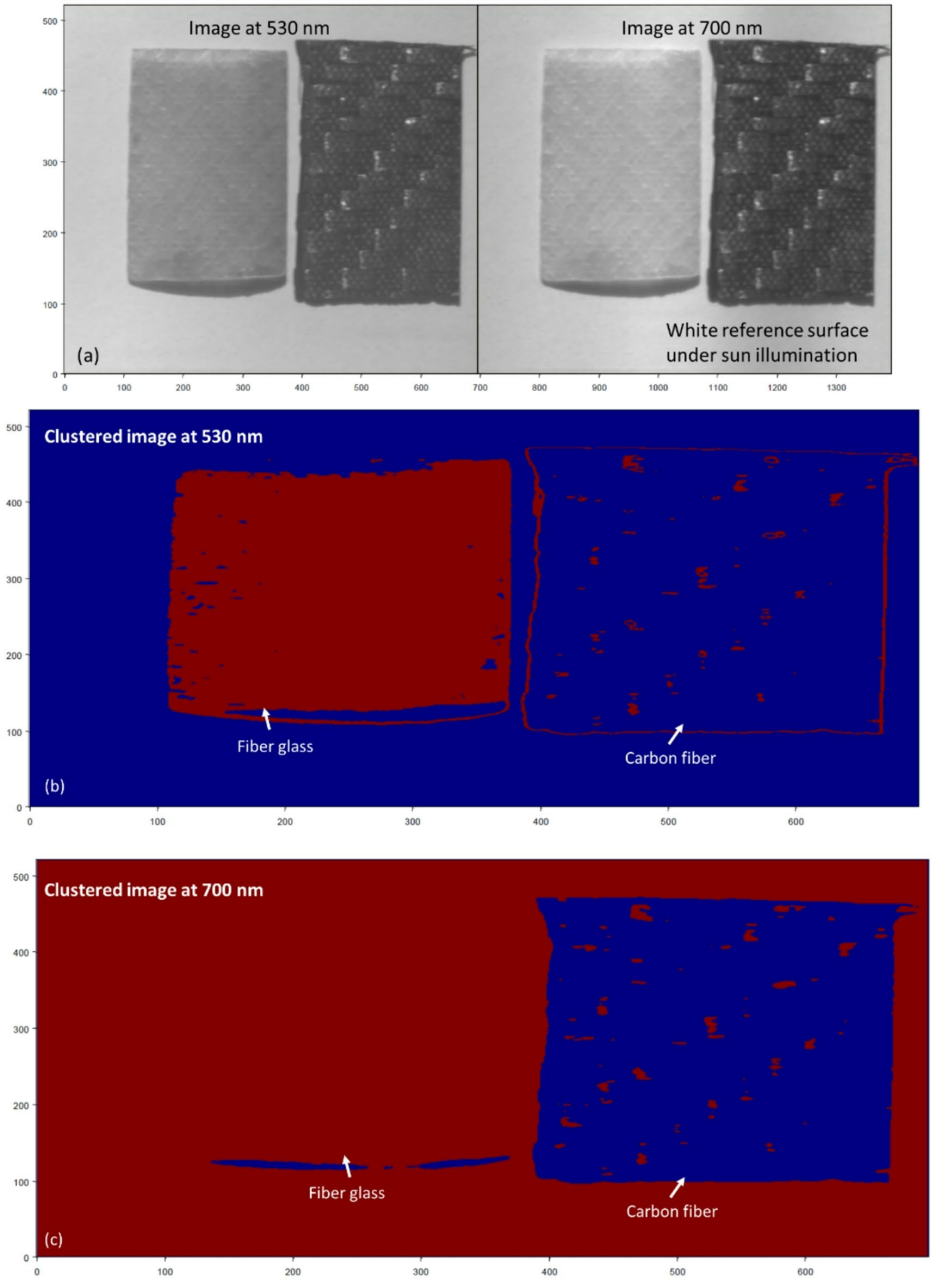
(**a**) HS images of GFRP and CFRP samples captured at 530 nm and 700 nm against a high-reflectance white reference background under direct solar illumination. (**b**) K-M clustering result at 530 nm, successfully isolating the glass fiber composite. (**c**) K-M approach outcome at 700 nm, successfully isolating the carbon fiber composite.

To further assess the robustness and real-world applicability of the proposed methodology, we extended the experiments to include two more challenging scenarios. In the first scenario, we replaced the neutral black background with a high-reflectance white reference surface to simulate daylight reflections and increased ambient interference. As shown in Fig. 16, K-M clustering applied to HS images at 530 nm and 700 nm still achieved clear separation of GFRP and CFRP despite the brighter background. In the second scenario, we substituted the halogen lamp with direct sunlight to mimic field-operational conditions. Figure 17 demonstrates that, even under solar illumination, the proposed HSI + K-M clustering workflow reliably classifies the two materials at both key wavelengths, confirming the approach’s adaptability to diverse outdoor environments.

According the outcomes in Figs. 14, 16 and 17, This work highlights the robustness and precision of the proposed method in identifying the material composition of UAV structures, even when traditional detection methods are ineffective. By utilizing HS imaging combined with the proposed clustering technique, we demonstrated that it is possible to accurately classify UAV materials based on their unique spectral signatures, even in complex scenarios involving unknown, painted, or coated objects. The successful classification of the unknown UAV sample underscores the practical applicability of our approach for real-world UAV detection and material identification.

## Conclusion

In conclusion, this study introduces an innovative approach to unmanned aerial vehicles (UAVs) detection and classification, focusing on the material composition of UAV structures through hyperspectral imagery and K-Means (K-M) clustering. With the widespread adoption of UAVs across industries, the need for advanced detection methods that extend beyond shape and size becomes critical, especially in security-sensitive contexts. Traditional detection systems often struggle to distinguish UAVs from other objects or to classify them based on structural material, which is crucial for accurate identification and effective response strategies. The proposed method leverages the SOC710 hyperspectral imager and a broad-spectrum light source to capture the unique diffuse reflectance characteristics of carbon fiber-reinforced polymers (CFRP) and glass fiber-reinforced polymer (GFRP) composites, key materials in UAV construction. By utilizing K-M clustering and a moving average filter, we successfully classify these materials, identifying CFRP at 700 nm and GFRP at 530 nm, with high precision and reliability. This material-specific approach to UAV detection not only enhances accuracy by reducing false positives but also addresses limitations in conventional systems, offering a powerful tool for differentiating UAVs based on their spectral signatures. The research holds significant potential for security, surveillance, and infrastructure protection, where understanding UAVs material composition is essential for effective detection and mitigation. Our findings underscore the value of hyperspectral imagery combined with advanced clustering as a robust solution for UAV detection and material classification in diverse operational scenarios.

## Data Availability

The authors stated and declare that all the datasets used and/or analyzed during the current study are available from the corresponding author on reasonable request to preserve the copyright. The authors stated and declare that all code exists and is available.
